# The Potential Use of Resveratrol for Cancer Prevention

**DOI:** 10.3390/molecules24244506

**Published:** 2019-12-09

**Authors:** Dominique Vervandier-Fasseur, Norbert Latruffe

**Affiliations:** 1 Team OCS, Institute of Molecular Chemistry of University of Burgundy (ICMUB UMR CNRS 6302), Univ. Bourgogne Franche-Comté, 21000 Dijon, France; dominique.vervandier-fasseur@u-bourgogne.fr; 2 Team Bio-PeroxIL, “Biochemistry of the Peroxisome, Inflammation and Lipid Metabolism” (EA7270)/Univ. Bourgogne Franche-Comté/Inserm, 21000 Dijon, France

**Keywords:** cancer, prevention, resveratrol, approach strategies, mechanisms, innovative formulations

## Abstract

In addition to the traditional treatments of cancer and cancer prevention, the use of natural compounds, especially those found in food, should be considered. To clarify if resveratrol has the potential for cancer prevention and the possibility of use in therapy, the following must be taken into account: data from epidemiology, clinical protocol (case and control), preclinical studies (lab animals), use of established cell lines as models of cancer cells, test tube assays (enzymes activities), and requirements of nanotechnologies in order to discover new drugs to fight cancer. From this perspective and future expected advances, more information is needed such as improved efficacy, methods of application, and the synergistic sensitization of resveratrol as an adjuvant. In addition, resveratrol nanoformulation is considered to overcome its weak bioavailability.

## 1. Introduction

This review focuses on the potential of resveratrol for cancer prevention and begins with the fate of cancer, i.e., the morbidity and mortality in the world due to cancer and the different types of cancer. Then, treatments and prevention of cancer are considered, especially, whether resveratrol has the potential for cancer prevention and could possibly be used for cancer therapy.

Regarding molecular interactions within selected groups of the population, the following are emphasized: the data obtained from epidemiology, clinical studies, preclinical studies, the use of cell lines as models, the assays on test tube enzymes, and the use of nanoparticles to hopefully discover new treatments to fight cancer. From these experimental procedures, the main aspects are developed as follows: identifying pathways of antitumor mechanisms of resveratrol, innovative formulations of resveratrol, perspectives, and conclusions. On the basis of tumor cell lines, the mechanisms of antiproliferative and pro-apoptotic activities of resveratrol have included: pro-oncogenic and tumor suppressor miRNAs expression modulation; NBkB, PPAR, PGC1α, NRF1,2, p53 transcription factors, and TGFβ signaling targeting pathways; prodifferentiating properties; and the synergistic effect of resveratrol on anticancer chemical drugs. In order to increase the poor bioavailability of resveratrol, which is a weakness for its future use, attention has been given to the development of nanoparticles that can transport and target resveratrol to cancer cells, as well as research on the antiproliferative properties of resveratrol metabolites.

## 2. The Fate of Cancer in the World

**Figure 1:**
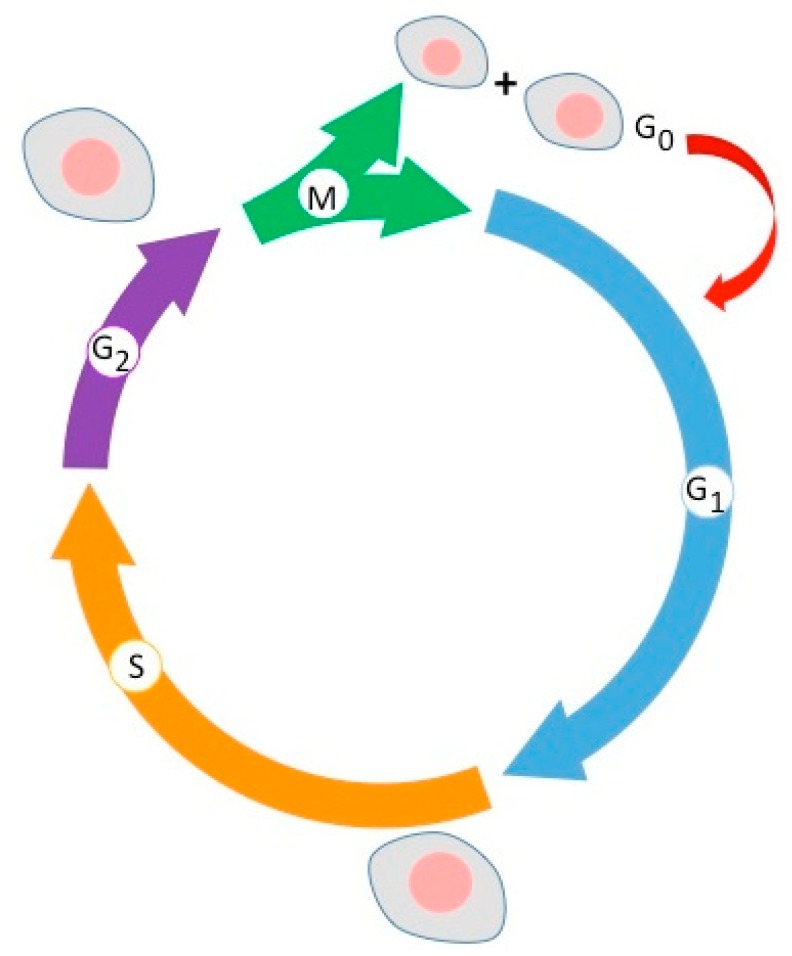
Brief recall of the cell cycle phases.

Basically, the mitosis cycle (Figure 1) of a cell is usually well controlled either at the G_0_ state (resting cell) or at the G1/S or G2/M phase checkpoints (cycling cell). 

**Figure 2:**
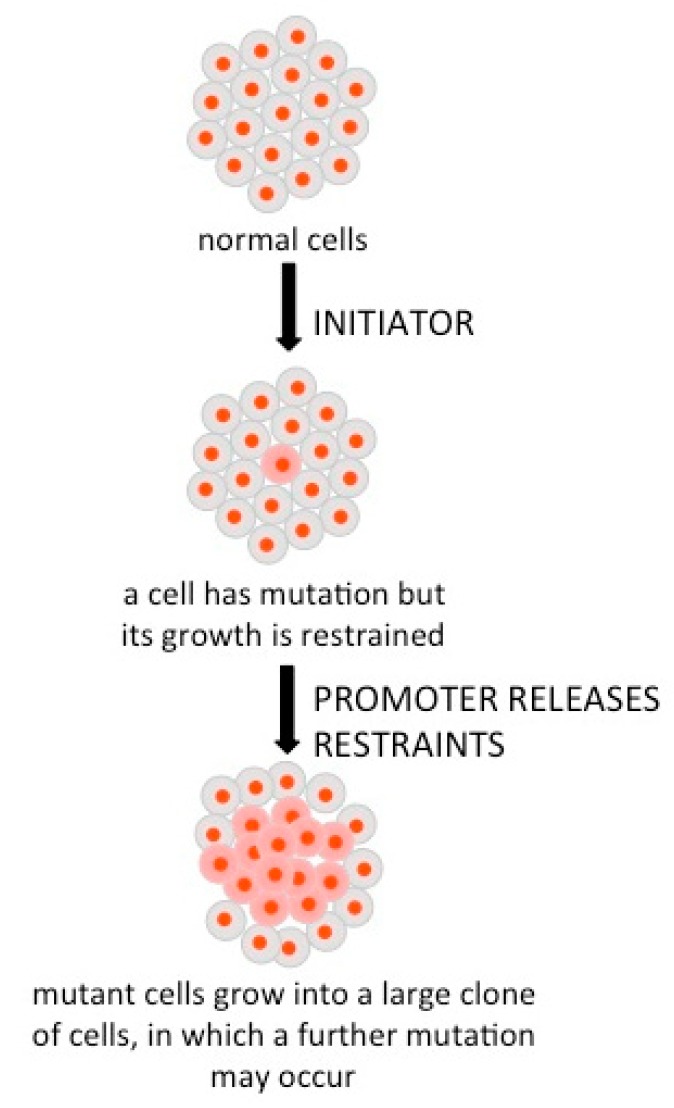
Initial steps leading to cancerous cells [1] (p. 15).

Nevertheless, in some unregulated conditions cells start to cycle continuously which can be the launch of a cancer process (Figure 2) [1] (p. 15). This event can be due to DNA mutations or to the overexpression of oncogenes under a favorable metabolic environment (growth factors, oxygen, and glucose). A normal cell becomes cancerous after three steps (initiation, promotion, and progression) to establish a localized tumor, which can be subsequently disseminated into new tumors (metastasis). 

## 3. Different Types of Cancer

The probability of a man or a woman unfortunately getting cancer during their lifetime is estimated to be around 0.5 and 0.3, respectively. Each year, there are around 14.1 billion new cases of cancer (7.4 billion men and 6.6 billion women) and more than 8.2 billion deaths (4.6 billion men and 3.5 billion women), which is the second cause of mortality. In Europe, in men, lung cancer is the first cause of mortality (410,000) followed by colorectal cancer (447,000) (second) and prostate cancer (417,000) (third); in women, breast cancer (464,000) is followed by colorectal cancer (second) and lung cancer (third) [2].

## 4. Treatments and Prevention of Cancer

In cancer therapy, the traditional treatments are surgery, chemotherapy, radiotherapy and, if relevant, immunotherapy, either as single treatments or successively. Prior to cancer treatment, there are ways to avoid the development of a tumor. It has been reported that food and nutrition contain keys factors for the development or decrease of cancer risk, where 30% to 40% could be avoided with prevention measures [1]. Interestingly, epidemiological, clinical, preclinical, and experimental studies demonstrate the beneficial effect of diet (especially Mediterranean diet) on digestive cancers. Fibers, polyphenols (including resveratrol), and omega-3 fatty acids appear to be the most protective components.

## 5. Why Choose Resveratrol?

**Figure 3:**
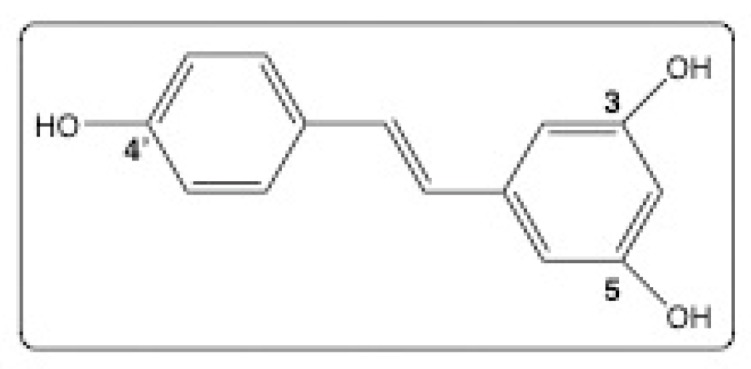
Structure of trans-resveratrol RSV.

Historically, the preventive effect of resveratrol towards cancer, especially skin and mammal cancers in mice, started with the discovery of Pezzutto’s group in 1997 [3]. Resveratrol (RSV) or trans-3,4′,5-trihydroxystilbene (Figure 3) is a natural polyphenol found in large quantities in the root of the Japanese knotweed (*Polygonum cuspidatum*) (Table 1) [4].

RSV is also produced in large amounts by vine plants in response to biotic infections (for instance *Botrytis cinerea*) but also synthesized by reacting to abiotic stresses [9]. Due to this, RSV is a vine phytoalexin that stimulates the natural defense of grape plants. RSV is also produced by other edible plants, such as hops [6], peanuts (Table 1), as well as by numerous berries (blackberries, blackcurrants, blueberries, mulberries and cranberries) [5,7,8].

Due to its antioxidant properties, RSV provides numerous beneficial effects to humans including the prevention of not only cancer but also cardiovascular diseases, neurodegenerescence, and low-grade inflammation. In addition, RSV improves mice longevity and physical activity through the sirtuin pathway [10,11,12,13,14].

The cell absorption of RSV follows both the diffusion process and facilitated transport [15]. Then, in the hepatic cell, RSV is largely metabolized [16] as glucurono, sulfo- and tauto-conjugates, and three hydrophilic forms of RSV to be easily eliminated by MRP-1 [17]. The blood transport of RSV involves binding to albumin and lipoproteins [18]. The usual threshold of in vitro resveratrol effect is between 20 and 50 micromolars. Interestingly, Aires et al. [19] have shown that 3-*O*-sulfate-RSV, a metabolite of resveratrol, was able to inhibit human colon cancer cell lines by induction of DNA damages, apoptotic process, and accumulation of cells in S-phase. Moreover, the mixture of this metabolite with two others (i.e., 3-*O*-glucuronide-RSV and 4′-*O*-glucuronide-RSV) induced a synergistic effect.

## 6. Experimental Approaches of the Problem

To address the topic of the potential of resveratrol to prevent cancer, the following questions should be addressed: What do we learn from epidemiological and from clinical studies? What information do animals and cultured cell protocols and enzyme assays provide to explain mechanisms? And, Is the challenge of seting up new preparations relevant for innovative purposes in order to improve RSV efficiency?

### 6.1. Epidemiology

In 2005, Levi et al. showed that resveratrol from grape consumption is inversely related to the risk of breast cancer, as reported in a study carried out on 369 cases vs. 602 controls of Swiss women followed from 1993 to 2003 [20].

Renaud et al. [21] conducted an analysis on 34,014 middle-aged men obtained from a comprehensive health appraisal from 1978 to 1983 in the eastern part of France. They concluded that a moderate intake of wine was associated with a 20% reduction in all-cause mortality by cancer. From this result, wine polyphenols, especially RSV, were considered to be a major protecting agent.

Adherence to a Mediterranean diet, with the major basis ingredients of polyunsaturated fatty acid, polyphenols from olive oil, and polyphenols from grape, including resveratrol, decreased the risk of developing head and neck cancer [22].

In 2014, Semba et al. [23] reported a prospective cohort study that was carried out from 1998 to 2009 in two villages in the Chianti area on a population-based sample of 783 community-dwelling men and women 65 years or older. They concluded that resveratrol levels did not show a significant effect on mortality risk, notably by cancer.

### 6.2. Clinical Studies (Case Control Studies)

A phase I, randomized, double-blind pilot study of micronized resveratrol (SRT501) in patients with hepatic metastases showed that cleaved caspase-3, a marker of apoptosis, significantly increased by 39% in malignant hepatic tissue following SRT501 treatment as compared with tissue from the placebo-treated patients [24].

A study conducted on healthy volunteers suggested that repeated administration of high doses of resveratrol generates micromolar concentrations of the parent molecule and much higher levels of glucuronide and sulfate conjugates in the plasma. It was concluded that the observed decrease in circulating IGF-I and IGFBP-3 might contribute to cancer chemopreventive activity [25].

Twenty patients with histologically confirmed colorectal cancer consumed eight daily doses of resveratrol at 0.5 or 1.0 g before surgical resection. Levels of resveratrol and its metabolites were consistently higher in tissues originating in the right side of the colon as compared with the left side of the colon. Consumption of resveratrol reduced tumor cell proliferation by 5% (P = 0.05). The authors suggested that daily p.o. doses of resveratrol at 0.5 or 1.0 g were sufficient to elicit anticarcinogenic effects [26].

### 6.3. Preclinical Studies

It has been shown that resveratrol induced a 40% drop in lung metastasis in rats and mice [27]. The association between pterostilbene and quercetin inhibited the metastatic activity of B16 melanoma cells in mice liver [28]. Resveratrol delayed the development and reduced the metastasizing capacity of spontaneous mammary tumors in the lungs of HER-2/neu protooncogene transgenic mice [29]. Red wine extracts prevented preneoplastic foci density in chemically induced intestinal tumors in mice [30].

### 6.4. Cell Lines

Various human tumor-established cell lines have been used to evaluate the effect of resveratrol and elucidate its action mechanism, including cell lines from the following: colorectal (SW480 cell line) [31], intestine (Caco2 cell line), liver (HepG2 hepatic derived cell line) [32], lymphoma [33], prostate (PCa cell line) [34], glioma cells [35] and tumoral cardiac cells [36].

Resveratrol, as well as red wine extracts, inhibit SW480 cells. The effect is dependent on the concentration in these extracts. In this model the cell uptake in resveratrol is enhanced by quercetin [30]. The metabolism of resveratrol is modulated by red wine extracts. In these cells there is a decrease in conjugation extent as compared with those in hepatic-derived cells. Resveratrol displays a pro-apoptotic effect on SW480 cells [37]. Interestingly, resveratrol shows pro-differentiating effects on skeletal muscle stem cells [38].

### 6.5. Test Tube Enzymes and Modeling

The direct effect of resveratrol on enzyme activities or its binding to receptors has been studied, reporting inhibition of cyclo-oxygenases 1 and 2 (COX 1,2), two pro-inflammatory enzymes [39]. The sirtuin-1 deacetylase activity involved in apoptosis is activated by RSV [40]. The αvβ3 integrin receptor has been shown, by modeling, to bind resveratrol and some derivatives [41]. From this, RSV inhibits human myeloma cell proliferation via crosstalk between the integrin αvβ3 receptor and the IGF-1 receptor [42].

## 7. Identified Pathways of Antitumor Mechanisms of Resveratrol

Antigenotoxic effects of resveratrol have been reported in HL60 colorectal cell line and in mice [43]. Conversely, in 2012, Heger et al. showed that the intake of a resveratrol-containing dietary supplement had no impact on the DNA stability in healthy subjects [44].

After cell exposure, RSV is accumulated in lipid rafts, which is the first step for endosome formation and apoptosis triggering [37,45]. The following signaling molecules, which are transcription factors involved in the cell cancer promotion or prevention, have been reported to be sensitive to resveratrol: PPAR, PGC1α, NFkB, NRF1,2, and p53 [46]. The nucleus cell cycle components, phosphatases, and cyclins are targets of RSV [47]. In addition, resveratrol triggers apoptosis signaling pathways [48].

RSV modulates pro-oncogenic or the expression of tumor-suppressor miRNAs. Indeed, previously, we have shown that miRNAs, non-coding small RNAs, are factors of the resveratrol-mediated tumor suppressor activity in the SW480 colon cancer cell line. More precisely, resveratrol treatment decreases the levels of several oncogenic miRNAs targeting genes encoding tumor suppressors and effectors of the TGFβ signaling pathway, while increasing the levels of miR-663 targeting TGFβ1 transcripts. While upregulating several components of the TGFβ signaling pathway, resveratrol decreases the transcriptional activity of SMADs, the main effectors of the canonical TGFβ pathway [49]. In addition, miRNAs appear to be new signaling molecules of resveratrol in mediating anti-inflammatory effects in THP-1 monocyte, where resveratrol upregulates miR-663, a miRNA potentially targeting multiple genes implicated in the immune response. MiR-663 decreases endogenous AP-1 activity and impairs its upregulation by LPS, at least in part, by directly targeting Jun B and Jun D transcripts. The downregulation of AP-1 activity by resveratrol is miR-663 dependent, and the effects of resveratrol on both AP-1 activity and Jun B levels are dose-dependent. Moreover, resveratrol impairs the upregulation of miR-155 by LPS in a miR-663-dependent manner [50]. In addition, miRNAs are involved in resveratrol mediated C_2_C_12_ myoblast differentiation. Indeed, resveratrol initiates the early steps of skeletal muscle undifferentiated cells C_2_C_12_ in a myoblast state into an ongoing differentiated stage as myotube. Essential tissue specific transcription factors are modulated by resveratrol through putative miRNAs (myf 5, myod1 by miR-20b and srf by miR-133) [38].

## 8. Seeking Innovative Formulations of Resveratrol

The poor aqueous solubility and the weak bioavailability of RSV are great drawbacks of this natural and no toxic polyphenol which prevent its clinical trials. Numerous resveratrol derivatives have been synthesized with the aim of modifing physico-chemical features and improving biological activities of the parent polyphenol [51]. However, unfortunately, examination of the therapeutic potency of the parent resveratrol molecule could not be achieved because of its solubility issues. However, the resveratrol nanoformulation concept, developed for thirty years, has kept unmodified structurally RSV and has provided promising results that have been the subject of several reviews [52,53,54]. RSV has been loaded into liposomes [55], cyclodextrins [56], solid lipid nanoparticles [57], mesoporous silica nanoparticles [58], or metallic nanoparticles [59]. The results from several studies are in agreement, i.e., satisfactory RSV-loadings by different types of nanoparticles, stabilization of the trans isomer thanks to the protection of resveratrol against UV, improved bioavailability due to increased aqueous solubility of resveratrol, and therefore antioxidant and antitumoral activities are enhanced due to the higher RSV concentration close to the therapeutic targets [53]. However, problems of release and targeting of RSV are dependent on its metabolism as free RSV [52]. Several in vitro studies have highlighted that the size and shape of nanoparticles encapsulating RSV play an important role in biochemical mechanisms, in addition to the RSV biological action. Because the size of solid lipid nanoparticles (SLN) is smaller than 180 nm, RSV-loaded SLN can pass through keratinocyte membranes without causing fundamental changes in cell morphology and metabolic activity. In contrast, a high concentration of RSV-loaded SLN, and therefore free RSV, are released around the nuclei and polyphenol plays its cytostatic role [57]. Ultradeformable vesicles are also good candidates to promote drug permeation through the skin because their highly deformable membrane allows them to penetrate through skin pores smaller than vesicles [60]. Thereby, in an ex vivo study, it was shown that RSV and 5-fluorouracil-loaded ultradeformable liposomes could penetrate and accumulate in the deep porcine skin layer; from the skin deposit, both drugs were gradually released and acted as an antitumoral drug for 5-fluorouracil and an antioxidant agent for RSV to promote apoptosis [61].

Targeting the improvement of a drug is one of the challenges in nanoformulation. In the case of RSV, different strategies have been considered. For example, the presence of dequalinium (DQA), a mitochondrion-tropic molecule, at the surface of PEG_2000_-DSPE, a lipid material used in stealth liposomes, warrants the mitochondria targeting in cancer cells. The intracellular and mitochondria uptakes of RSV-loaded DQA PEG_2000_-DSPE liposomes have been highlighted in both non-resistant A549 lung cells and resistant A549/cDDP lung cells. Therefore, RSV delivered inside the mitochondria has triggered cell apoptosis through the mitochondria pathway [55].

Transferrin (Tr) is a natural ligand of transferrin receptor overexpressed in glioblastoma, a highly aggressive cancer with a poor prognosis. Its grafting on RSV-loaded liposomes affords an effective targeting towards brain cancer cells [62]. Indeed, regarding free RSV or RSV-liposomes, RSV-loaded-Tr-liposomes showed a higher antiproliferative activity on U-87 MG cells, a human glioblastoma cell line. While liposome and transferrin play the role of carrier and cell target, respectively, RSV is really responsible for the cytotoxicity and apoptosis processes. In addition, and in a promising way, RSV-loaded-Tr-liposomes could inhibit U-87 MG tumor growth in nude mice more effectively than free RSV or RSV-liposomes [62].

The incorporation of a cancer-targeting ligand directly into nanoparticle structures is another way to promote drug release more selectively to cancer cells. Thereby, nanoparticles have been obtained by coupling human serum albumin (HSA), a non-toxic carrier of weakly soluble drugs and folic acid (FA) a cancer-targeting ligand [63]. Antitumoral activities of RSV-loaded HSA-FA nanoparticles were evaluated in in vitro assays of human liver cancer HepG2 cells and in in vivo experiments on H22 tumor-bearing mice. These studies have shown that the combination of HSA and RSV, on the one hand, slowed down the release of the drug at the injection site, and on the other hand, significantly increased the RSV accumulation in the tumor regarding RSV-loaded HSA nanoparticles without folic acid as a targeting agent. In addition, bioavailability of RSV-loaded HSA-FA nanoparticles turned out to be six times higher than the free polyphenol and no toxic effects of RSV-loaded HSA-FA nanoparticles were detected on healthy rat organs. In contrast, the decrease in size of the tumor was not mentioned [63].

The therapeutic potential of RSV-nanoparticles has been highlighted by numerous studies conducted on various cancer cell lines and tumors. At the core of nanoparticles, RSV keeps all of its health benefits, which are released closer to the tumor thanks to the carrier. In addition, the presence of targeting agents on the nanoparticle wall or inside the nanoparticle structure undeniably increases antitumoral activities of RSV-loaded nanoparticles.

RSV is well-known for its non-toxic character. In contrast, toxicity studies addressing the combination of RSV-nanoparticle are lacking and subsequently slow down preclinical and clinical trials. In addition, it remains difficult to consider switching the syntheses of RSV-nanoparticles from a laboratory-scale to a large-scale production because of the high cost of its implementation [64]. However, the high number of possible combinations among the nature, size, and shape of the nanoparticles, as well as the nature and incorporation methods of targeting agents leads to therapeutic considerations of RSV-encapsulated nanoparticles.

## 9. Perspectives

Synergistic sensitization properties of RSV with various drugs have been highlighted. Resveratrol plays the role of an adjuvant with pro-apoptotic drugs (CH11 and Trail) and with 5-fluorouracil (5-FU) caspases 3. A cell death marker is activated in chemoresistant HT29 colon tumor cell line by CH11 and Trail after resveratrol sensitization [48]. Resveratrol sensitizes SW480 colorectal tumor cell line to 5-FU [65]. Interestingly, metabolites of resveratrol also synergize with chemotherapeutic drugs to induce cell death [19].

Interestingly, there are synergies between resveratrol and some other polyphenols found in wine, such as quercetin and catechin [66,67,68]. These synergies between grape polyphenols could overcome the poor bioavailability of resveratrol as confirmed by the high urinary level of resveratrol in humans. The synergistic effect of resveratrol, with other wine polyphenols in the prevention of pathologies, supports the efficacy despite the limited plasmatic level of single wine polyphenol.

In addition to grapes which are the major source of food from vine plant, the by-products of grape vines (wood, leaves, roots) also contain high content of polyphenols, in particular, resveratrol which can be used as a supplement in food [69]. For better retention of RSV, it is recommended that it should be taken in an amphiphilic environment, for example, oil cooked sauces, and even some alcohol (resveratrol from red wine, in moderation).

The main role played by resveratrol in the stimulation of natural defenses has been confirmed by the appearance of resistance of different transgenic plant species resulting from the overexpression of the stilbene synthase gene [70]. Currently, laboratory-made edible plants producing resveratrol already exist. They are obtained by introducing the stilbene synthase gene, such as found in tomatoes [71], apples [72], papayas [73], and other species (lettuce, cauliflower, yeasts, and bacteria). Combined with the natural antifungal properties of resveratrol, one could envision a better preservation of fruits and vegetables by spraying with a resveratrol-based preparation, subsequently delaying aging. Unfortunately, due to the high cost of resveratrol from vines, Chinese resveratrol extracted from *Polygonum cuspidatum* as well as its synthetic counterpart could become economically profitable.

## 10. Conclusions

Resveratrol shows all of the antiproliferative and pro-apoptotic properties to be an antitumor agent. However, these effects are mostly obtained in vitro or with small animals at concentrations that are too high to be compatible with its low availability in vivo. Fortunately, this molecule is not toxic and can be used as a food supplement at high concentrations that are compatible with a significant plasmatic level. Moreover, the synergistic effect of other food polyphenols and the fact that resveratrol plasmatic metabolites also exhibit antiproliferative activities is relevant for diet-dependent cancer prevention [19]. These findings have been recently confirmed by Sankaranarayanan et al., who reported that 2,4,6-trihydroxybenzoic acid, a flavonoid metabolite, is itself an antiproliferative agent [74]. Although the improvement of therapies remains an essential challenge, it is also very important to concentrate on prevention, especially nutritional prevention.

## Figures and Tables

**Table 1:** The trans-resveratrol concentrations determined in different sources. Adapted from L. Le Corre, PhD thesis 2005 [5].

Natural Sources	Trans-RSV Concentration (μg/g)	References
Hops	0.50 ± 0.05	[6]
Peanuts	5.10 ± 2.85	[7]
Peanut butter	0.30 ± 0.10	[7]
Grape skin	27.50 ± 1.30	[8]
Itadori root (*Polygonum cuspidatum*) Ko-jo-kon	523 ± 1	[7]
